# Experiences of unemployed and/or work-disabled cancer survivors who have pursued to return to paid employment: a focus group study

**DOI:** 10.1007/s11764-024-01657-5

**Published:** 2024-08-14

**Authors:** M. A. Greidanus, F. van Ommen, A. G. E. M. de Boer, P. Coenen, S. F. A. Duijts

**Affiliations:** 1 Department of Public and Occupational Health, Amsterdam UMC Location University of Amsterdam, Meibergdreef 9, Amsterdam, The Netherlands; 2 Amsterdam Public Health Research Institute, Societal Participation and Health, Amsterdam, The Netherlands; 3 Cancer Center Amsterdam, Cancer Treatment and Quality of Life, Amsterdam, The Netherlands; 4 Department of Public and Occupational Health, Amsterdam UMC Location Vrije Universiteit, De Boelelaan 1117, Amsterdam, The Netherlands; 5 Amsterdam Movement Sciences Research Institute, Musculoskeletal Health, Amsterdam, The Netherlands; 6 Department of Research & Development, Netherlands Comprehensive Cancer Organisation (IKNL), Rijnkade 5, Utrecht, The Netherlands; 7 Department of Medical Psychology, Amsterdam UMC Location Vrije Universiteit, De Boelelaan 1117, Amsterdam, The Netherlands

**Keywords:** Cancer survivors, Employment, Focus groups, Job application, Motivation, Neoplasm

## Abstract

**Purpose:**

To explore experiences of unemployed and/or work-disabled cancer survivors who have pursued to return to paid employment.

**Methods:**

Four digital focus group interviews were conducted with 16 cancer survivors (< 10 years post-diagnosis) who have pursued to return to work within the last 2 years. Interview topics included motivations, facilitators of and barriers to job seeking, and returning to and maintaining paid employment. Interview audio recordings were transcribed verbatim and analyzed using conventional content analyses.

**Results:**

Participants were mostly female (94%), and the majority had successfully returned to paid employment (56%). Both intrinsic factors (e.g., sense of purpose, social interactions) and extrinsic factors (e.g., financial necessity) motivated their return to paid employment. During job seeking, participants experienced facilitators including support, personal qualities (e.g., life experience), and trial workplaces. Barriers included inadequate support, perceived employer discrimination, and work ability uncertainty. Returning to and maintaining employment was facilitated by flexible work, supportive colleagues, and intrinsic drive, while barriers included side effects (e.g., fatigue) and overly demanding work.

**Conclusions:**

Unemployed and/or work-disabled cancer survivors are generally motivated to return to paid employment by both intrinsic and extrinsic factors, but uncertainty about their ability and inadequate support may hinder this. These findings highlight the need for trial workplaces, support during every phase of return to paid employment, and a flexible, supportive workplace.

**Implications for Cancer Survivors:**

Tailored interventions addressing the needs identified in this study are urgently needed. The recommendations provided offer strategies for various stakeholders to enhance support for unemployed and work-disabled cancer survivors.

**Supplementary Information:**

The online version contains supplementary material available at 10.1007/s11764-024-01657-5.

## Introduction

Improved long-term cancer survival rates have made it increasingly important to focus on life after cancer. For cancer survivors in the working population, employment is one of the main pillars of cancer survivorship [1]. Being able to work not only enhances the quality of life of cancer survivors but also contributes to financial stability and social well-being [2–5]. However, many survivors suffer from persistent adverse effects stemming from both cancer and its treatments, such as fatigue, depression, anxiety, and concentration problems, impacting their work ability and work participation [6–9]. Such impairments may persist over time and continue to affect cancer survivors’ long-term work ability [10]. Overall, about one-third of cancer survivors are unable to return to their previous employment [11, 12]. Compared to a non-cancer population, cancer survivors are 1.4 times more likely to be unemployed [13].

Compared to cancer survivors returning to their previous employment, unemployed and/or work-disabled survivors seeking new paid employment often lack the opportunity to gradually assess and improve their work ability [14]. These survivors encounter distinctive challenges in their job search, including apprehension about applying for positions, disclosing their cancer history, and potential employer reluctance stemming from a possibly overestimated risk of cancer recurrence [14–16].

The challenges of unemployment and work disability extend beyond the individual cancer survivor, affecting employers and society at large as well. The non-participation of numerous unemployed and/or work-disabled survivors, who are skilled individuals motivated to work, signifies a loss for employers, particularly in times of labor market scarcity [17, 18]. Additionally, the societal burden associated with unemployment and disability benefits underscores the importance of promoting strategies to enhance work participation of cancer survivors who are motivated to return to paid employment [19, 20].

Existing research predominantly focuses on return-to-work (RTW) interventions tailored for cancer survivors who are currently employed [10]. However, there is a scarcity of interventions specifically designed for unemployed and/or work-disabled survivors, and the available evidence regarding the effectiveness of these interventions is limited [9]. To develop such interventions, it is essential to gain a comprehensive understanding of the experiences of unemployed and/or work-disabled cancer survivors seeking to re-enter the workforce. Exploring their motivations, barriers, and facilitators encountered during various phases of returning to or maintaining paid employment, can inform future interventions tailored to the needs of this population. Therefore, the aim of this study was to explore experiences of unemployed and/or work-disabled cancer survivors who have pursued to return to paid employment.

## Methods

### Study design

A focus group study was conducted with cancer survivors who had engaged in job-seeking activities for paid employment within the past 2 years at the time of recruitment. Approval of the Executive Board of the Medical Ethics Committee of the Amsterdam UMC, location Academic Medical Center (AMC) was not necessary, since the burden for participants was low and their psychological integrity was not at stake. Informed consent was obtained from all participants. Data collection took place in October 2021. To enhance explicit and complete reporting of this study, the “Consolidated Criteria for Reporting Qualitative Research” (COREQ) checklist was used (Online Resource 1) [21].

### Participants and recruitment

Participants were included in this study if they met the following criteria: (1) being diagnosed with cancer within the past 10 years, (2) aged between 18 and 65 years, and (3) have engaged in job-seeking activities for paid employment in the past 2 years, originating from a situation of unemployment and/or work disability (either solely unemployed or unemployed and partly or fully work-disabled). Job-seeking activities may include, among others, searching for and responding to vacancies and participating in job interviews. As our study aimed to encompass a diverse range of experiences related to seeking, returning to, and maintaining paid employment, both cancer survivors who returned to paid employment and those who did not were eligible to participate.

Potential eligible cancer survivors were recruited via social media platforms (e.g., LinkedIn and cancer-related Facebook groups) and a Dutch online cancer platform (www.kanker.nl). Interested cancer survivors indicated willingness to participate via a registration form. After telephonic screening for eligibility by one of the first authors (M.G.), eligible cancer survivors received an invitation containing focus group details, an informed consent form, a questionnaire seeking descriptive information, and a pre-paid return envelope. Before the focus groups took place, interested and eligible survivors were asked to complete and return the consent form and questionnaire.

Recruitment took place until 16 cancer survivors were included. These numbers were expected to lead to data saturation based on a previous focus groups study with a similar aim [14]. Data saturation on a specific topic (i.e., motivations, job-seeking experiences, and experiences related to returning to and maintaining paid employment) was considered achieved when no new themes emerged on that topic in the final focus group session, a process known as inductive thematic saturation [22]. All participants received a gift voucher of 25 euros in gratitude for their participation.

### Data collection

Data were collected via a short self-reported questionnaire and focus groups. The questionnaire gathered data regarding participant characteristics, including gender and age, and health- and work-related variables including diagnosis, treatment, current employment status, and recent job-seeking activities undertaken by the participant. The 90-min focus group sessions were organized using video conferencing (Microsoft Teams), as many participants preferred digital communication due to the ongoing impact of the COVID-19 pandemic. We aimed to organize focus groups with four participants per session, as we consider this number to be conducive to fostering discussions during video conferencing focus groups. To promote the recognition of each other’s experiences and facilitate meaningful exchange and discussion during the focus groups, our aim was to group participants homogeneously based on whether they did or did not return to paid employment (i.e., homogeneous group based on their current work status). The focus groups were led by one of the first authors (M.G.), an experienced male moderator with a PhD degree, who had no prior relationship with the participants before the study. One or two other authors (F.v.O., or F.v.O. and S.D.) were present as observers during the focus group sessions. The focus groups commenced with a succinct introduction of the goals and outline of the focus groups by the moderator, followed by the attendees introducing themselves. Following that, pre-specified topics were addressed: (1) the participants’ motivation behind returning to paid employment, (2) their experiences related to job seeking, and (3) their experience related to returning to and maintaining paid employment (Table 1). The moderator provided participants with an opportunity to individually write down their initial thoughts on a topic for 3 min (i.e., their perceived motivators, facilitators, and barriers). Subsequently, open-ended questions were posed by the moderator to stimulate discussions on these topics. All focus groups were audio-recorded. No field notes were taken during or after the focus groups.

**Table 1:** Topic list interviews

Topic	Example questions*
*Motivation behind returning to paid employment*	What prompted you decision to return to paid employment?
*Experiences related to job seeking*	What facilitators and barriers have you experienced while seeking paid employment? This includes activities such as searching for suitable vacancies, responding to vacancies, and participating in job interviews
*Experiences related to returning to and maintaining paid employment*	What facilitators and barriers have you experienced when returning to paid employment? What facilitators and barriers have you experienced while maintaining paid employment?

^*^For every question, multiple alternative and probing follow-up questions were posed to achieve a comprehensive understanding of the topics

### Data analysis

Data gathered from the self-reported questionnaires were analyzed using descriptive statistics via SPSS (version 28.0; IBM Corp., Armonk, NY). The focus group interviews were transcribed verbatim by F.v.O. and two research assistants. Thereafter, two authors (M.G. and F.v.O.) independently performed a conventional content analyses using MAXQDA qualitative data analysis software (VERBI Software GmbH, Marburg, Germany) [23]. Firstly, both authors assigned codes reflecting the different topics of interest (i.e., motivations and facilitators and barriers per phase of returning to paid employment) to the transcripts and categorized them into groups that closely reflected the content of the original text, employing open coding within the topics of interest. Following this, the groups were clustered to capture overarching themes. Both authors performed these two steps independently to enhance reliability. Afterwards, the themes, groups, and codes were compared; in cases of disagreement, conflicts were discussed until consensus was reached. No follow-up interviews or focus groups were carried out, nor were the final codes or themes returned to the participants for review, to minimize participant burden.

## Results

Sixteen cancer survivors participated, of which the majority were female survivors (*n* = 15; 94%) with a primary diagnosis of breast cancer (*n* = 11; 69%) (Table 2). On average, participants had been diagnosed with cancer 4 years prior to study inclusion (range, 2–8 years), and most of them were either employed full-time or part-time at the time of diagnosis (*n* = 14; 87%). At the time of inclusion, nine participants were employed by an employer (56%), eight were assessed as either fully or partially work-disabled (50%), and six were actively seeking paid employment (38%), with these categories not being mutually exclusive. Most of the participants had been involved in searching for job vacancies (*n* = 12; 75%), applying to job vacancies (*n* = 13; 81%), and attending job interviews (*n* = 12; 75%) since their cancer diagnosis. Since the participants were recruited through open invitations, no characteristics of the non-participating group of cancer survivors, nor the reasons for non-participation, were available. The focus groups lasted on average 110 min (range, 93–137 min), excluding a 10–20-min break. Table 3 presents the major themes identified across the three topics. Data saturation was achieved for the topics of motivations for returning to paid employment and experiences related to seeking paid employment. However, saturation was not achieved for the topic of returning to and maintaining paid employment, as new themes on this subject emerged in the final focus group.

**Table 2:** Participants’ characteristics

Participant characteristics (*n* = 16)		*n* (%)	Mean (range)
Sociodemographic variables			
Age			48 (24–58)
Gender	Female	15 (94)	
Level of education	Secondary education Intermediate vocational education Higher professional education	0 (0) 5 (31) 11 (69)	
Health-related variables			
Primary diagnosis	Breast cancer Lymphoma/leukemia Other (uterus cervix, pancreatic, vulvar)	11 (69) 2 (13) 3 (19)	
Time since diagnosis			4 (2–8)
Treatment*	Surgery Chemotherapy Radiation Hormone treatment Immunotherapy	13 (81) 13 (81) 9 (56) 5 (31) 4 (25)	
Work-related variables			
Work status at diagnosis	Full-time for an employer Part-time for an employer Unemployed Student	4 (25) 10 (63) 1 (6) 1 (6)	
Current work status*	Full-time for an employer Part-time for an employer Fully work-disabled Partly work-disabled Other (i.e., volunteer work)	1 (6) 8 (50) 5 (31) 3 (19) 1 (6)	
Actively in pursuit of paid employment	Yes	6 (38)	
Job-seeking activities since diagnosis*	Job searching Applying to job vacancy Open application Job interview Other (i.e., volunteering, networking, reintegration agency guidance, guidance from coach of Employee Insurance Agency, becoming self-employed, entering a therapeutic workplace, education via subsidy program)	12 (75) 13 (81) 4 (25) 12 (75) 7 (44)	

^*^Multiple options may apply to one participant

**Table 3:** The major themes across the three topics

Perceived motivators behind returning to paid employment	Experiences in seeking paid employment	Experiences in returning to and maintaining paid employment
*Intrinsic motivators* • A sense of purpose • Social interactions • Structure in their lives • Financial autonomy • Being a “normal” person • Personal development	*Facilitators:* • Assistance in assessing work and functional abilities • Work-related support • Personal qualities • A trial workplaces or volunteer work • A favorable work market • The possibility to work from home	*Facilitators:* • Flexibility (i.e., concerning workplace, working hours, and job tasks) • Supportive colleagues • Intrinsic drive • Standing up for yourself • Being flexible and adaptive
*Extrinsic motivators* • Financial necessity • Pressure from the Social Security Agency • Social pressure	*Barriers:* • Suboptimal support from the Social Security Agency • Discrimination from potential employers • Societal norms (e.g., no gap in resume) • Lack of a suitable workplace • Financial reasons (e.g., possible setback in benefits) • Uncertainty • Cancer treatment and its side effects	*Barriers:* • Consequences of cancer and its treatment (e.g., fatigue and difficulties with multitasking) • No knowledge and understanding among colleagues • Overly demanding work • Struggling with work-life balance

### Motivation behind returning to paid employment

A variety of intrinsic and extrinsic factors motivated participants to return to paid employment. Regarding intrinsic factors, participants highlighted *a sense of purpose* in their lives as a major driving force. This sense of purpose, as mentioned by the participants, could encompass a feeling of usefulness, either for themselves, for others, or for society at large.

“*You [the participant] wish to make a contribution to society. The thought of merely staying at home made me feel somewhat down. Yes. It simply doesn’t provide me with the feeling of being particularly valuable….*” (#15: f, 55 y, employment: yes).

Participants also linked this sense of purpose with the feeling of being part of society.

“*I thought, then [when not in paid employment, resp.] I would really start to become isolated. I am someone who enjoys being around people. For me, the social aspect is also important*.” (#13: f, 47 y, employment: yes)

Participants also emphasized the role of paid employment in fostering *social interactions*. The social circles of various participants gradually diminished, influenced in part by their children moving out and their family and friends being occupied with daytime work. Engaging in paid employment emerged as a means for them to fulfill the need for social interactions.

“*It’s just really nice to [...] to meet new people again. And, yes, new things, new stories, new experiences. [...] I’m always quite open to new connections and, yeah, I just really enjoy being socially active instead of sitting at home*.” (#15: f, 55 y, employment: yes)

In addition, for many participants, the desire for *structure in their lives* also motivated them to return to paid employment. Having paid employment offers them a concrete incentive to get up in the morning.

“*I’m not much of a ‘morning person.’ So, when I go to work, at least I have sort of a daily routine, and I have to set my alarm on time. When I wasn’t working, I found that challenging; I had to create my own daily routine*.” (#5: f, 53 y, employment: yes)

In some instances, the participants viewed it as essential for their own sense of well-being to achieve *financial autonomy*, being financially independent from, for instance, a partner or their social benefit.

“*I have three daughters and I want to be an example and show that as a woman, you also need to be independent and have your own income. Not being dependent on your partner.*” (#5: f, 53 y, employment: yes)

Participants also expressed the need to move beyond the identity of a patient. According to their perspective, retuning to paid employment could contribute to *simply being a “normal” person* again.

“*If I look deep within myself, it’s also about wanting things to be just normal again. I want to move away from where I am now, so to speak.*” (#4: f, 51 y, employment: no)

Finally, some participants felt an intrinsic motivation to pursue *personal development*, encompassing both intellectual growth and career advancement. They believed that paid employment could play a significant role in achieving this.

“*You also want, at least that’s how I feel, to continue developing. […] to still undergo a sort of learning process. And the workplace is a very suitable place for that.*” (#1: f, 50 y, employment: no)

Regarding extrinsic factors, *financial necessity* was a major reason for participants to return to paid employment. For certain participants, the income from social benefits was insufficient to cover expenses.

“*I simply had the necessity for income because I don’t have someone beside me whose salary I can partially rely on, so to speak.*” (#3: f, 48 y, employment: yes)

Related to this, some participant’s experienced *pressure from the Social Security Agency*, which provides unemployment and work disability benefits, to return to paid employment, for example, when the re-evaluation of the participant’s work ability was imminent, potentially affecting the participant’s benefits.

“*And it was also, well, the question was whether my symptoms would be considered sufficient grounds for [social benefits].*” (#3: f, 48 y, employment: yes)

“*The Social Security Agency breathing down your neck, you’re practically forced to [...] apply for everything, even for positions you don’t want to pursue.*” (#8: f, 52 y, employment: no)

Finally, some participants mentioned experiencing *social pressure* from friends and family to return to paid employment.

“*There is still a bit of feeling like a freeloader. Even though I know I genuinely have complaints that warrant receiving social benefits. Still, it feels to me that others sometimes see it that way. So, I don’t tell many people that I receive [social benefits].*” (#3: f, 48 y, employment: yes)

### Facilitators of and barriers to job seeking

Participating cancer survivors identified support as a crucial factor facilitating their success in job seeking. This support could encompass *assistance in assessing their work and their functional abilities*.

“*I had a really great coach there, who held up a mirror to me, saying, ‘Hey, you’re really pushing your limits. Maybe you shouldn’t do that right now.’ ... I found it quite annoying at first [...] But looking back, I realize that balancing work and home life is genuinely manageable now.*” (#13: f, 47 y, employment: yes)

Participants also mentioned that *work-related support* from career coaches or reintegration specialists played a pivotal role in achieving positive job-seeking experiences.

“*[The career coach] really made me reflect on what I can do, what I enjoy about work, what I still think I can achieve, and what I genuinely want. Yes, he helped me get all of that sorted out….*” (#13: f, 47 y, employment: yes)

The participants identified several key facilitators during the job-seeking period which encompassed their *personal qualities*, such as a strong professional network, life and work experience, assertiveness, self-confidence, authenticity, and a drive for achievement.

“*Experience plays a vital role - something that applies to each of us. The fact that you have so much experience, not just in the workplace but also in life, I consider that a success factor.*” (#5: f, 53 y, employment: yes)

For some participants, engaging in *trial workplaces* or *volunteer work* served as progress towards paid employment, thus serving as a facilitator for the job-seeking phase. These settings enabled them to improve their skills, gradually reintegrate into work, expand their professional network, and demonstrate their capabilities to potential employers. Most importantly, such opportunities allowed cancer survivors to test or confirm their work abilities.

“*I would need [a trial workplace] to even discover [my own work ability].*” (#6: f, 55 y, employment: no)

Additionally, a *favorable job market* was cited by participants as a facilitator for successful job seeking. Lastly, in the light of the trend of remote work prompted by the COVID-19 pandemic, one participant noted that the *possibility to work from home* was also a facilitator.

“*You can better schedule breaks at home [when working from home], [...] it’s attractive to alternate it [working from home] with office work. And during a job interview, it’s much easier to bring up these points.*” (#3: f, 48 y, employment: yes)

Next to these facilitators, participating cancer survivors encountered various barriers to successful job seeking. These barriers can be categorized into external barriers (i.e., related to anything but the cancer survivor) and internal barriers (i.e., related to the cancer survivor themselves). Concerning external barriers to successful job seeking, participants mentioned *suboptimal support from the Social Security Agency*. This included a lack of tailored support, slow or unassertive responses from coaches, or a lack of connection with the coach.

“*[The support from the Social Security Agency] is quite rigid; when deviating from its structure, you... you fall outside of it, and that can make things challenging.*” (#16: f, 49 y, employment: yes)

“*We already have so little energy, and it takes so much energy to get anything done with them [the Social Security Agency]*.” That’s just really difficult (#8: f, 52 y, employment: no)

Participants also experienced possible *discrimination from potential employers* when disclosing their diagnosis during job seeking.

“*So, I thought, [...] let me include my illness in my resume. [...] But well, yeah, then I’ve gone to a second interview twice and then I feel like I already have that job in the bag and then I still get rejected. And why? I don’t know. And then, at some point, you start to think that it’s because of my illness.*” (#8: f, 52 y, employment: no)

Some participants also indicated that *societal norms* posed a hindrance during their job seeking. Having a gap on their resume or being of older age did not align with the ideal image.

“*Society as a whole is naturally oriented towards health, youth, and success, even though that’s not the reality. [...] being ill, experiencing sadness, or facing failure, are actually a part of [life]. Essentially, companies should be a reflection of society*.” (#5: f, 53 y, employment: yes)

Where trial workplaces pose a facilitator for job seeking, contrarily, the *lack of a suitable workplace* to assess work ability, or an inappropriate workplace, was mentioned as a barrier to job seeking.

“*I can apply, but which position should I apply for? One for 10 hours or 20? I have no idea if I can work more than 2 hours a day because I haven’t worked that long. I find that really difficult*.” (#6: f, 55 y, employment: no)

Another barrier to successful job seeking is the reluctance of participants to apply for jobs due to *financial reasons*. This is because their benefits could be reconsidered if they demonstrate their ability to work. This could potentially lead to a net setback in their financial situation.

“*I just really wish I had some kind of safety net to fall back on if the house of cards collapses again.*” (#1: f, 50 y, employment: no)

Finally, participants also mentioned overly rigid job vacancies (e.g., lacking flexibility in the number of working hours) and an unfavorable job market as obstacles to job seeking.

When it comes to internal barriers to successful job seeking, participants mentioned *uncertainty* as a primary obstacle. This uncertainty could pertain to their own work ability and workplace skills or uncertainty about their preferences.

“*You don’t really know what you’re committing to and what you can promise, both to yourself and to a potential employer.*”(#10: f, 37 y, employment: yes)

Lastly, many participating cancer survivors mentioned that their *cancer treatment and its side effects* served as major barrier during job seeking. The most cited impediments in this regard were low energy levels, difficulty with stimuli, visible physical changes, and cognitive problems such as forgetfulness.

“*But my limited energy and cognitive limitations just mean that I can’t start a job with 20 or 24 hours a week somewhere. [...] where will I end up if I apply somewhere? I have to do something completely different than what I’ve done before.*” (#4: f, 51 y, employment: no)

### Facilitators of and barriers to returning to and maintaining paid employment

Participating cancer survivors who returned to paid employment identified several work-related and personal factors that facilitated returning to and maintaining paid employment. *Flexibility* emerged as a key facilitator, encompassing options such as a flexible workplace (e.g., having the possibility to work remotely), flexible working hours, and adaptable job tasks.

“*I find it particularly important that I can do my work at moments and places that suit me. [..] if I don’t feel well one day, I can just postpone my work, and I can essentially work on a Saturday.*” (#3: f, 48 y, employment: yes)

Additionally, participants regarded *supportive colleagues* as a facilitator for returning to and sustaining employment.

“*[A colleague and I] can divide the tasks between us. [...]. He has no trouble at all taking on those tasks that need to be done ad hoc and quickly. [...] So we find a symbiosis there in a very organic way.*” (#1: f, 50 y, employment: no)

Regarding personal factors, participants mentioned an *intrinsic drive* as a facilitator for returning to and maintaining paid employment. This intrinsic drive, as described by one participant, comprised two key determinants.

“*I do believe that my illness has indeed given me more determination. And in combination with the desire to prove myself, I did persevere and ultimately achieved success.*” (#2: m, 24 y, employment: yes)

Participants additionally emphasized the importance of *standing up for themselves*, setting their own boundaries (e.g., by being open and honest with their manager), and concurrently, *remaining flexible and adaptive* to different situations.

“*I also had trouble remembering things, so at work, I carry a notepad and jot things down. [...] I have to find little tricks for everything to be able to do my job well.*” (#16: f, 49 y, employment: yes)

The identified barriers of returning to and maintaining paid employment were primarily related to the *consequences of the participants’ cancer and its treatment*. Fatigue, difficulties with multitasking, memory impairments, limited and unpredictable work ability, and a low tolerance for sensory overload were all limiting consequences. One participant explained how fatigue could unexpectedly overwhelm her.

“*[Fatigue] is often challenging to explain to people [...] it’s not a gradual thing, but rather like someone extinguishing a candle right at that moment. And then you’re empty.*” (#12: f, 55 y, employment: no)

Participants mentioned that it is important for colleagues to have a certain *level of knowledge about cancer and understanding of the situation*. A lack of this was experienced as a barrier to returning to and maintaining paid employment, as explained by one participant.

“*I have explained once that ‘I have limited capacity, and traveling also drains my energy,’ but then a week later, someone schedules a meeting from 9 to 4, which is both too early [...] and too long. [...] I don’t think it’s a deliberate attempt to bother you, but somehow, it’s challenging to get through to people.*” (#3: f, 48 y, employment: yes)

Certain work characteristics hindered participating cancer survivors in returning to and maintaining paid employment. Firstly, *overly demanding work*, such as excessive work pressure from the outset, was perceived as a potential barrier.

“*[I needed a place] where I could gradually adjust, experience, get the time to acclimatize, to see how things would go, to try things out, but it had to go full throttle right away, like going 200%. And that, that just didn’t work.*” (#12: f, 55 y, employment: no)

Participants also considered it a hindrance when they had to work below their skill level, when they had to take training on the job, and when the employer was unwilling to make job accommodations. Lastly, an important barrier experienced by participating cancer survivors was their *struggle with maintaining their work-life balance*.

“*I really struggled to find the balance between my personal life and work. [...] I find myself constantly assessing the days ahead. [When I anticipate busy days], then I know I need to pause and take it easy. It’s challenging.*” (#16: f, 49 y, employment: yes)

## Discussion

In this focus group study, we found that both intrinsic factors (e.g., sense of purpose, social interactions) and extrinsic factors (e.g., financial necessity) motivated the return to paid employment of cancer survivors. Support from, e.g., career coaches or colleagues, the availability of trial workplaces, and flexible work arrangements were identified as facilitators across the various stages of returning to paid employment. Conversely, a lack of support, uncertainty regarding their own work ability, and consequences of cancer and its treatment were among the identified barriers.

### Interpretation of findings

This study identified both intrinsic and extrinsic motivators for returning to paid employment among unemployed and/or work-disabled cancer survivors. According to self-determination theory [24], extrinsic motivation, such as financial necessity, is not inherently negative [25]. What is crucial is that individuals understand the underlying reasons for returning to work and feel autonomously motivated to do so [25]. However, extrinsic motivation often involves external control, such as that exerted by the Social Security Agency. Previous research on unemployed individuals without a history of cancer indicated that those under such external control more often experience negative emotions, such as feelings of worthlessness, presumably because external control is accompanied by stressful and pressuring experiences [26, 27]. Furthermore, the more these individuals feel compelled to search for a job, the less likely they are to take proactive steps to return to paid employment [26]. Intrinsic motivation is the most autonomous type of motivation, as individuals naturally and freely pursue their interest [25], which is in this case, returning to paid employment. In contrast to externally controlled actions, experiencing autonomy in the decision and actions to be taken to return to paid employment is positively related to well-being and job search intensity [26, 27]. Therefore, fostering intrinsic motivation to return to paid employment and autonomy in doing so appears to be more beneficial for job satisfaction and actual reemployment than relying on extrinsic motivation with high external control.

We found distinct experiences encountered by unemployed and/or work-disabled cancer survivors in seeking and starting paid employment, compared to existing studies focused on those who return to their previous employment. Firstly, unemployed and/or work-disabled cancer survivors may face heightened uncertainty regarding their own abilities, including challenges in assessing their work capacity and identifying suitable jobs opportunities and hours. Initiating a new job and finding accommodating employers can be particularly challenging, as many strategies aimed at addressing functional limitations and symptoms require workplace adjustments, such as gradually increasing the workload [10, 15, 28]. Secondly, unemployed and/or work-disabled cancer survivors often lack a supportive work environment. Previous research indicated that a supportive work environment, and more specifically feeling supported by colleagues, is associated with positive RTW outcomes [2, 4, 29–31]. Lastly, unemployed and/or work-disabled cancer survivors encounter discrimination when disclosing their diagnosis, resulting in reduced employment opportunities compared to employed cancer survivors [16]. From the perception of employers, this discrimination could stem from concerns about whether cancer survivors are able to meet job demands and be productive, as well as concerns about the impact on workplace culture, organizational costs, and absenteeism [16, 32]. Considering these findings, we emphasize the necessity for tailored interventions for unemployed and/or work-disabled cancer survivors, specifically addressing the increased uncertainty, lack of supportive work environment, and discrimination they encounter in seeking and starting paid employment.

### Strengths and limitations

The strengths of our study lie in capturing the experiences of both cancer survivors who succeeded in re-entering the labor market, and those who did not, thus facing challenges in the process. This approach allowed us to capture both experiences, facilitators, and barriers faced by cancer survivors in their attempts to return to paid employment, as well as the ongoing barriers and facilitators in maintaining paid employment. Additionally, we only included participants who had recently sought new employment, minimizing potential recall bias in their experiences. Another strength is that we achieved data saturation regarding motivations for returning to paid employment and experiences related to seeking paid employment, which enhances the reliability and generalizability of our results concerning these topics.

A limitation of this study is that we did not achieve data saturation on the topic of returning to and maintaining paid employment. The last focus group identified some new themes related to this topic, which introduces uncertainty regarding the completeness of the results in this area. The lack of data saturation is likely due to the limited number of participants who had actually reached the phase of returning to and maintaining paid employment, as not all participants had secured paid employment at the time of the study. Therefore, the number of participants who shared their experiences with this phase is lower compared to the phase of job seeking. Additionally, due to the feasibility constraints of the study, we were unable to organize additional focus groups to achieve data saturation on this theme. A second limitation is the low representation of male participants, with only one male participant included, and the overrepresentation of breast cancer survivors. Consequently, the extent to which the findings fully represent the experiences of male cancer survivors and non-breast cancer survivors is unclear. However, as breast cancer is one of the most prevalent types of cancer among working-age patients, the number of breast cancer patients in our study can be considered representative. Lastly, the reliability of the results could have been further enhanced by sending the final codes or themes to the participants for review. However, we chose not to do this in order to minimize the participation burden on this vulnerable group of cancer survivors.

### Recommendations for future research

To advance our understanding of supporting unemployed and/or work-disabled cancer survivors in their return to paid employment, future research should focus on several key areas. Firstly, interventions tailored to their identified needs should be developed and evaluated for effectiveness and cost-effectiveness. By systematically addressing the challenges faced by these individuals, interventions can enhance their chances of successfully returning to paid employment. Additionally, investigating the sustainability of intrinsic versus extrinsic motivation in guiding the return to paid employment is crucial. Understanding the long-term impact of these motivational factors can inform strategies for promoting sustainable employment outcomes for cancer survivors and guide intervention design. Furthermore, exploring potential gender and diagnosis differences in the experiences of cancer survivors returning to paid employment is essential for improving tailored interventions. Lastly, future research should further explore the experiences of unemployed and/or work-disabled cancer survivors in returning to and maintaining paid employment.

### Recommendations for practice

Based on the identified facilitators and barriers, we compiled an overview of the specific practical needs of unemployed and/or work-disabled cancer survivors in their process of re-entering the labor market:

1. The need for workplaces that enable cancer survivors to assess their work ability and establish relationships with potential employers. This can be achieved through trial workplaces or volunteer work, where cancer survivors can gain firsthand experience in returning to work while expanding their professional network. Acquiring work experience in such environments may lead to sustainable employment.
2. The need for support throughout all phases of job seeking, returning to, and maintaining paid employment. During the job-seeking phase, the focus should be on identifying roles that align with the work ability of cancer survivors, taking into consideration the effects of cancer and its treatments. Upon returning to and maintaining paid employment, it is essential to provide a supportive work environment that acknowledges the challenges faced by cancer survivors, such as coping with demanding job tasks or managing short- and long-term consequences of their diagnosis. Monitoring a healthy work-life balance during this phase is also vital to ensure the sustainability of their return to paid employment. This comprehensive support requires coaches who can address both work-related issues and aid in assessing the work and functional ability of cancer survivors.
3. The need for flexible and supportive workplaces. Employers should provide flexibility in terms of working hours, workplace location (e.g., telecommuting if necessary), and work tasks. This allows cancer survivors to tailor their duties to their individual abilities and effectively manage the consequences of cancer. Moreover, it is crucial for colleagues to be willing to lend assistance when certain tasks are challenging for the cancer survivors. By incorporating these elements, workplaces can foster a supportive and inclusive environment for cancer survivors, facilitating their sustainable reintegration into the labor market.

## Conclusions

This focus group study provides valuable insights into the experiences of unemployed and/or work-disabled cancer survivors as they strive to re-enter the labor market. By delving into their motivations for work, as well as facilitators and barriers encountered during various phases of re-entering paid employment, we have developed an elaborate understanding of their journey towards returning to paid employment and the sustainability of such employment. The findings emphasize the necessity of trial workplaces, where cancer survivors can assess their work ability and establish relationships with potential employers. Additionally, the study highlights the importance of tailored support throughout the various phases of returning to paid employment to enhance successful workforce reintegration. Moreover, the identified need for flexible and supportive work environments highlights the significance of accommodating the unique needs of these cancer survivors. The recommendations stemming from this study provide actionable strategies for policymakers, employers, and researchers to enhance the support provided to cancer survivors. By integrating these recommendations into tailored interventions, stakeholders can contribute to creating inclusive and supportive work environments, enhancing the well-being and quality of life of cancer survivors.

## Supplementary Information

Below is the link to the electronic supplementary material.

Supplementary file1 (DOCX 29 KB)

## Data Availability

Data is available from one of the first authors (MG) upon reasonable request.
